# A perturbation-based balance training program for older adults: study protocol for a randomised controlled trial

**DOI:** 10.1186/1471-2318-7-12

**Published:** 2007-05-31

**Authors:** Avril Mansfield, Amy L Peters, Barbara A Liu, Brian E Maki

**Affiliations:** 1 Institute of Medical Science, University of Toronto, Toronto, Ontario, Canada; 2 Centre for Studies in Aging, Sunnybrook Health Sciences Centre, 2075 Bayview Ave, Toronto, Ontario, M4N 3M5, Canada; 3 Department of Medicine, Sunnybrook Health Sciences Centre, Toronto, Ontario, Canada; 4 Department of Medicine, University of Toronto, Toronto, Ontario, Canada; 5 Department of Surgery, University of Toronto, Toronto, Ontario, Canada

## Abstract

**Background:**

Previous research investigating exercise as a means of falls prevention in older adults has shown mixed results. Lack of specificity of the intervention may be an important factor contributing to negative results. Change-in-support (CIS) balance reactions, which involve very rapid stepping or grasping movements of the limbs, play a critical role in preventing falls; hence, a training program that improves ability to execute effective CIS reactions could potentially have a profound effect in reducing risk of falling. This paper describes: 1) the development of a perturbation-based balance training program that targets specific previously-reported age-related impairments in CIS reactions, and 2) a study protocol to evaluate the efficacy of this new training program.

**Methods/Design:**

The training program involves use of unpredictable, multi-directional moving-platform perturbations to evoke stepping and grasping reactions. Perturbation magnitude is gradually increased over the course of the 6-week program, and concurrent cognitive and movement tasks are included during later sessions. The program was developed in accordance with well-established principles of motor learning, such as individualisation, specificity, overload, adaptation-progression and variability. Specific goals are to reduce the frequency of multiple-step responses, reduce the frequency of collisions between the stepping foot and stance leg, and increase the speed of grasping reactions. A randomised control trial will be performed to evaluate the efficacy of the training program. A total of 30 community-dwelling older adults (age 64–80) with a recent history of instability or falling will be assigned to either the perturbation-based training or a control group (flexibility/relaxation training), using a stratified randomisation that controls for gender, age and baseline stepping/grasping performance. CIS reactions will be tested immediately before and after the six weeks of training, using platform perturbations as well as a distinctly different method of perturbation (waist pulls) in order to evaluate the generalisability of the training effects.

**Discussion:**

This study will determine whether perturbation-based balance training can help to reverse specific age-related impairments in balance-recovery reactions. These results will help to guide the development of more effective falls prevention programs, which may ultimately lead to reduced health-care costs and enhanced mobility, independence and quality of life.

## Background

Falling is a leading cause of serious injury, loss of independence and nursing-home admission in older adults [1]. Previous research investigating exercise as a means of falls prevention in older adults has shown mixed results. A number of studies have shown that exercise can help to prevent falls [2-11]; however, other exercise studies have shown little or no benefit [12-15]. Although exercise programs that include a balance-training component have tended to be most effective [2], no previous studies have targeted specific aspects of balance-recovery reactions. This lack of specificity may explain, in part, the failure to demonstrate a more pronounced reduction in fall rates.

It is important for fall-prevention interventions to include a focus on balance-recovery reactions, because it is the ability, or inability, to respond effectively to a balance perturbation ('loss of balance') that ultimately determines whether or not a fall occurs. Balance perturbations can arise from events such as slips, trips and collisions, but also occur as a consequence of volitional movement (e.g. turning, bending, reaching). Change-in-support balance reactions, which involve very rapid limb movements (stepping, or reaching to grasp an object for support), play a critical role in responding to balance perturbations (for recent reviews, see [16-19]). These compensatory stepping and grasping reactions are the only line of defence against large perturbations, but are also frequently recruited at lower magnitudes of perturbation (provided that subjects are permitted to react naturally, i.e. with no instructional or physical constraints on limb movement) [20,21].

A number of studies have reported age-related impairments in compensatory stepping reactions. Compared to young adults, older adults show increased frequency of collisions between the swing foot and stance leg [22], reduced step length [23], increased frequency of multiple-step responses [22-25], and an increased tendency to follow an initial forward or backward step with one or more lateral steps [24]. Few studies have examined age-related differences in compensatory grasping reactions; however, it has been reported that older adults tend to rely more than younger persons on arm reactions to recover balance [22], yet the speed at which they are able to initiate and execute grasping reactions is reduced [25]. Importantly, it appears that many of the age-related impairments in change-in-support reactions listed above are associated with increased risk of falling, as determined retrospectively [26-28] and prospectively [25]. Such findings support the view that training to promote more effective change-in-support reactions may help to reduce the risk of falls, and hence should be included as part of a falls prevention program [29].

**Figure 1:**
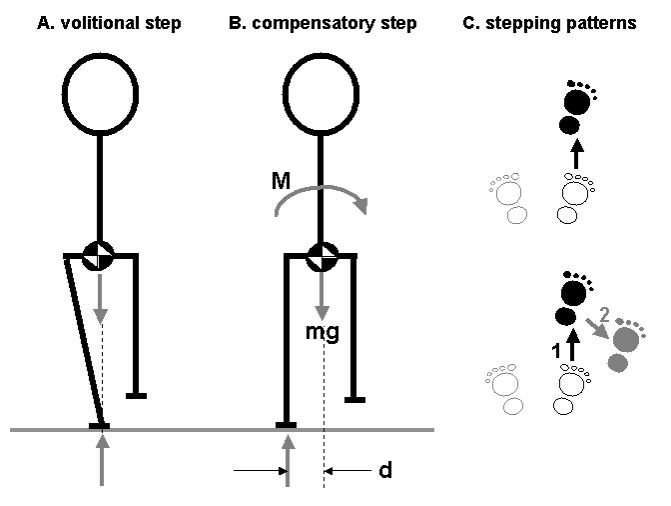
**Control of lateral stability during forward and backward steps**. Volitional steps are preceded by an anticipatory postural adjustment (APA) that acts to preserve lateral stability during the step by propelling the centre of mass toward the stance leg prior to lifting of the swing foot, thereby countering the tendency of the body to fall toward the unsupported side during the execution of the step (panel **A**). Conversely, APAs are typically absent or severely truncated during compensatory steps; as a result, the centre of mass falls toward the unsupported side of the body during the swing phase [the body weight (mg) creates a destabilising moment of force M = mg * d (panel **B**)]. In older adults, inability to arrest the lateral motion of the centre of mass during the landing phase of the initial forward or backward step often leads to one or more 'extra' steps in the lateral direction, whereas young adults typically respond with a single step (panel **C**).

The neural control of volitional limb movements differs in some fundamental ways in comparison to reactions that are evoked by postural perturbation [18,30]. It can therefore be argued that the most effective approaches to training compensatory stepping and grasping will involve the use of perturbations. A clear example to support this view pertains to the control of lateral stability during forward and backward stepping (Figure 1). During volitional stepping, anticipatory postural adjustments (APAs) act to preserve lateral stability by shifting the centre-of-mass toward the stance leg before lifting the swing foot. In contrast, during compensatory stepping, these APAs are typically absent or severely truncated [31,32]. As a result, the body falls laterally toward the unsupported side during the step and this lateral falling motion must be arrested during the landing phase. Training volitional stepping might lead to improved control of the APA but would probably not improve control of lateral stability during compensatory stepping. Another example pertains to training of reaction speed. It has been shown that older adults are slower than younger subjects to lift the foot when stepping voluntarily but are equally as fast as the young when the stepping reaction is evoked by postural perturbation [33]; hence, training to improve the speed of volitional stepping movements may provide little or no benefit in terms of the ability to execute effective compensatory stepping reactions.

Recently, a small number of studies have begun to assess the efficacy and feasibility of perturbation-based balance training in older adults [34-39]. These training programs have shown potential to improve reaction time for volitional stepping and grasping movements [34,37]; however, improvements in volitional stepping and grasping may not translate to balance-recovery situations, for the reasons noted earlier. To date, only one study has examined effects of perturbation-based training on the control of compensatory stepping reactions. This study demonstrated increased step length and faster step initiation following training [35]; however, the study was limited to persons with Parkinson's Disease. To our knowledge, no studies have directly addressed the potential to use perturbation-based training to counter specific impairments in compensatory stepping related to normal ageing, rather than neurological disease. Furthermore, no studies have investigated the potential to use perturbations to train compensatory grasping reactions.

The objective of the present study is to develop and test a perturbation-based balance-training program to counter specific, previously-reported age-related impairments in change-in-support stepping and grasping reactions. We hypothesize that subjects who undergo the perturbation-based training program will show greater improvements in the ability to step and grasp to recover balance in comparison to control subjects who undergo flexibility and relaxation training. In this paper, we describe the development of the perturbation-based training program and lay out the protocol for a randomised controlled trial to evaluate the efficacy of this program.

## Methods – Part A

### Development of the perturbation-based training program

#### Method of perturbation

In the simplest terms, maintaining stable upright stance involves keeping the centre of mass (COM) of the body over the base of support (BOS) defined by the feet (and, in some situations, the arms). Hence, the essential defining feature of any balance perturbation is that it induces relative motion between the COM and the BOS. For stance perturbations, this relative motion can be induced by causing displacement of either the COM (e.g. via cable waist-pull systems) or the BOS (e.g. via motion platform systems). During gait, this relative motion can also be induced when the cyclic progression of the COM and BOS is disrupted due to perturbation of the BOS (e.g. due to a slip on a low-friction surface, a trip on an obstacle, or sudden acceleration or deceleration of a treadmill).

The various available perturbation methods may differ in a number of respects, such as the pattern of motion induced at specific joints, the evoked sensory inputs and the pattern of the early evoked muscle activation [40]. Nonetheless, these various methods all fulfil the fundamental biomechanical requirement (disruption of the COM-BOS relationship) and can often elicit postural reactions that are similar in many respects [40]. Hence, it is possible that the training benefits derived using one type of perturbation may generalise (at least to some extent) to the reactions evoked by other types of perturbations. This, however, remains to be established. If there is limited generalisability of training benefits, then it would be best to choose a perturbation method that closely emulates the perturbations that lead to loss of balance and falls in daily life; however, it is unlikely that this can be accomplished using any one single method of perturbation. The training perturbations would need to simulate both slips and trips, (report to occur in approximately 40–60% of falls in older adults [41-43]) as well as the sizeable proportion of falls that do not involve ambulation, (e.g. self-induced perturbation during leaning, turning, reaching or sit-stand transfer movements; support-surface motion that occurs when standing in a moving vehicle [41,43]).

**Figure 2:**
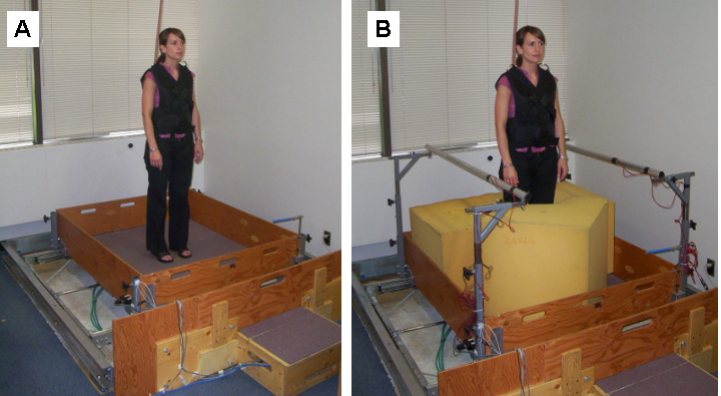
**Perturbation platform used during balance training**. Photographs of the training platform, configured for training of: **A. **stepping reactions, and **B. **grasping reactions. The surface of the platform is controlled to move 30 cm either forward, backward, left or right by means of pneumatic cylinders; the perturbation magnitude (platform velocity and acceleration) is altered by changing the pressure of the air delivered to the cylinders. A dual-axis accelerometer measures the magnitude and timing of the platform acceleration. During grasping training, handrails (equipped with force-sensing resistors to provide information about the timing of the reactions) are mounted on the platform, on one or both sides of the subject. These handrails are positioned at varying heights (87–101 cm) and distances from the subject (37–42 cm from midline) to simulate variability in handrail placement in daily life. Foam blocks are used to prevent foot movement and promote reliance on grasping reactions. A safety harness is worn at all times during training. (Image used with the consent of the model.)

For this study, we elected to develop a custom-designed pneumatic motion platform for the purpose of delivering the postural perturbations needed to evoke stepping and grasping reactions during training (Figure 2). One important feature of the moving-platform approach is the ease with which the direction of perturbation can be varied in an unpredictable manner [18]. Unpredictability is a critical requirement, as the CNS can learn to recognise any features of the perturbation that are predictable (direction, magnitude, waveform or timing), and can use this information to improve the efficacy of the balance reactions in a predictive manner [44-48]. This type of predictive control is unlikely to be helpful in responding to the unpredictable perturbations that commonly lead to falls in daily life; hence, the adaptations that result from training with predictable perturbations may have limited generalisability [18]. A second practical advantage of the motion-platform approach is that large numbers of perturbations can be delivered in a short span of time, hence maximising opportunity for training while minimising the risk of fatigue, which would be more likely to occur during gait-perturbation methods [36,39].

During the training program, the surface of the platform is controlled to move suddenly and unpredictably in one of four directions: forward, backward, left or right. The first session will be a familiarisation session, in which the minimum perturbation magnitude that requires a change-in-support response will be determined. During this initial session, subjects will be instructed to respond to the platform motion naturally. For the initial stepping training session, handrails will be mounted on the platform to help subjects feel at ease, but will be removed for subsequent sessions. Subjects are instructed to respond to the platform movement by stepping, if required (Figure 2A). For grasping training, a handrail is mounted on one or both sides of the subject and foam blocks are placed around the feet to prevent stepping (Figure 2B). Subjects will be instructed to recover balance by grasping the rail or rails "as quickly as possible" in response to the platform movement. Subjects will wear a safety harness secured to the ceiling at all times during the training.

#### Design of the program to optimise motor learning

The training program adheres to the principles of exercise prescription, that is, specificity, progressive overload, individualisation and random variation. In addition, we applied principles that dictate how feedback and instructions should be provided in order to optimise motor learning.

##### Specificity

**Figure 3:**
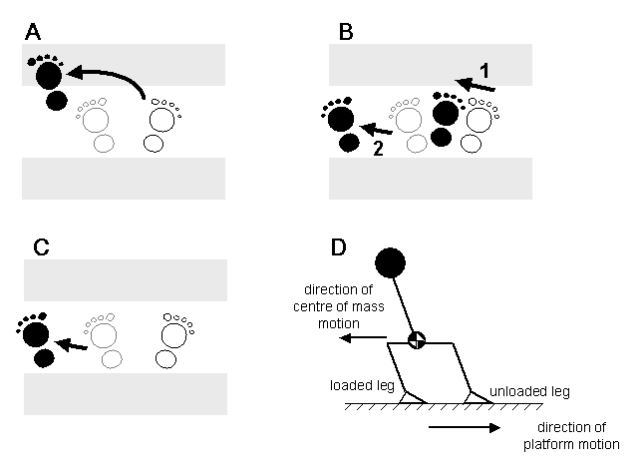
**Method to deter crossover steps during training**. Large foam blocks (indicated by the shaded areas) are placed in front of and behind the feet during the lateral-perturbation trials in the first two training sessions. Subjects cannot execute a crossover step because the foam prevents the swing foot from travelling or landing in front of or behind the stance foot (panel **A**). The training is intended to promote instead the use of a side-step sequence (panel **B**). Note that both of these step patterns involve an initial step with the foot that is unloaded as a result of the perturbation-induced body motion. The foam blocks also permit a lateral step with the loaded leg (panel **C**); however, this pattern of stepping tends to occur infrequently [22]. Note that panels **A-C **depict stepping responses evoked by rightward platform translation, which causes the subject to fall to the left; this motion of the body leads to an initial increase in the loading of the left foot and an unloading of the right foot (panel **D**). The natural tendency is to step with the unloaded leg.

The need to target specific goals is considered to be one of the most important motor training principles [49]. The perturbation-based program targets specific age-related impairments in compensatory stepping and grasping, as follows:

1. Multiple steps: Older adults take more steps to recover balance [22] and tend to respond to perturbations with a multiple-step response more frequently than younger adults [22-25]. Although use of multiple steps can be a pre-planned strategy in some situations [23], it appears that the 'extra' steps often emerge as a consequence of instability arising after the initiation of the first step [24]. Impaired ability to control the tendency of the centre-of-mass to fall laterally toward the unsupported side during step execution appears to be a particular problem, necessitating additional steps to recover lateral stability [24] (Figure 1C) as well as other step modifications [28]. The tendency to take multiple steps to recover balance predicts increased falling risk, and the tendency to follow an initial forward or backward step with a lateral step predicts an increased risk of falling laterally [25]. To reduce the frequency of multiple-step responses, subjects will be instructed to respond to the platform movement by taking as few steps as possible.

2. Foot collisions: Collisions between the swing foot and stance leg can jeopardise stability by impeding efforts to rapidly extend the base of support in the lateral direction [22]. Foot collisions are rare in young adults, but occur more frequently in older adults and are associated with increased risk of falling [22,25]. These collisions are most likely to have a serious effect on stability during prolonged crossover steps (Figure 3A). To train subjects to avoid such collisions, crossover responses will be discouraged and subjects will instead be guided toward using a sequence of side-steps (Figure 3B). During the first two training sessions, crossover responses to lateral perturbations will be discouraged by placing foam-rubber barriers in front of and behind the feet and instructing subjects "not to let your feet touch the blocks". During subsequent training sessions, the foam blocks will be removed and subjects will be instructed as follows: "if you need to move your feet, imagine the foam blocks were still there and try not to let your feet hit them".

3. Delay in onset of arm reactions and slowing of arm movement: Older adults show delays in the time taken to initiate and complete a compensatory grasping reaction, and this tendency is predictive of falling [25]. In order to train faster reaction and movement times for grasping reactions, subjects will be instructed to grasp the rails as quickly as possible. To further promote fast reactions, subjects will be provided with feedback about their response time using data from force-sensing resistors located on the handrails. There already exists some evidence that the latency of triggered reactions can be reduced with training [38,50]. Potentially, movement time could also be reduced. It is also anticipated that the training may lead to more effective grasping of the handrail, i.e. reduced incidence of errors, (e.g. overshoot, undershoot, collision of the hand with the rail) and more effective balance recovery (as evidenced by fewer "falls" against the safety harness).

4. Delayed attention switching: Balance-recovery reactions require attentional resources and cognitive processing [51,52]; however, older adults show delays in switching attention from ongoing cognitive or motor tasks to the task of balance recovery [53] and balance recovery is more attentionally demanding for older adults [51]. To improve ability to rapidly switch attention, cognitive and movement tasks will be included during training (Table 1). The type of task will be changed with each set of eight perturbations.

**Table 1:** Secondary tasks prescribed during perturbation-based training

**Type of ongoing task**^†^	**Description**
Repetitive movement of upper body	Reaching out in front (alternating hands) Reaching up (alternating hands) Reaching down (alternating hands) Alternately reaching up, out and down Reaching out to the side Trunk twists (side to side, keep head looking forward) Swinging arms (alternating), as if 'brisk walking'

Repetitive movement of lower body	Walking in place Shallow knee bends

Cognitive tasks	Words beginning with a letter (e.g. 'A': "apple, arrive") Words belonging to a category (e.g. 'girls names': "Mary, Anne") Counting backwards by 3's Backwards alphabet ("z y x w v u t......") Words that rhyme with a specified word (e.g. 'chair': "air, hair, fair") Multi-syllable word spelling (e.g. 'Mississauga') Subtracting 13, 17 or 19 from a number Talk about a topic (e.g. "tell me about the last book you read")

Held object^‡^	Plastic shopping bag Umbrella Book Handbag (women only)

^†^Type of task varies every eight perturbation trials.

^‡^For grasping training, subjects are asked to hold one of these items in their right hand and drop it in order to effectively grab the rail following the perturbation. These objects are also held for some sets during stepping training, but subjects are instructed to continue holding the object as there is no handrail nearby for them to grasp.

5. Ability to drop object to grab rail: The ability to effectively grasp a handrail may be impaired if an object is held prior to balance loss [54]. During some grasp-training trials, subjects will be given an object to hold (e.g. shopping bag, umbrella, book, handbag) and will be instructed to drop this object in response to the platform movement, in order to grab the rail.

##### Progressive overload

The perturbation-based training can be considered to represent an 'overload', which results in adaptation in the stimulated physiological systems. To elicit further improvement, there must be a progression in overload to further increase the challenge to the system [49]. Progressions will be made by increasing the magnitude of the applied perturbations whenever the subject is consistently able to recover balance by stepping or grasping without difficulty.

##### Individualisation

Training programs should be tailored to the individual needs and abilities of the participant [55]. Progressions should match the individual's rate of adaptation; therefore, progression in magnitude of perturbation will be based on the subject's ability to recover balance at the current magnitude.

##### Random, variable practice

Variability of practice conditions ensures that the learned motor skill will generalise to a wide variety of situations [56]. In order to optimise motor learning, the conditions should be varied in a random manner [57,58]. To facilitate skill acquisition during the initial stages of training (i.e. the first four sessions), antero-posterior and lateral perturbations will be presented in separate blocks of trials; however, in subsequent sessions, the direction of perturbation will be varied, trial-to-trial, in a pseudo-random sequence. To promote generalisability, the perturbations will be delivered while subjects perform a variety of cognitive and movement tasks, including a walking-in-place task that is intended to simulate some of the demands of executing the stepping and grasping reactions in response to perturbation of locomotion (Table 1).

##### Augmented feedback

Provision of external augmented feedback has been shown to improve performance of motor skills, but can hinder learning if the individual becomes too dependent on it [56]. To decrease dependence and facilitate learning, the feedback should be 'faded', or gradually reduced, as training progresses [59]. In this study, faded augmented feedback will be provided to improve the speed of the grasping reactions. Subjects will be told their handrail-contact timing score after every trial during the first week, after every second trial during the second week, etc. Augmented feedback will not be required for the step training, as pilot testing has shown that subjects are typically well aware of the occurrence of multiple steps and foot collisions during training.

##### Instructions

Instructions should direct the learner's attention toward important aspects of the skill being learned. However, to promote optimal learning, complex skills should not be broken down into smaller sub-components, and instructions should refer instead to the 'whole response' [59]. Furthermore, instructions that provide the learner with an external focus promote learning better than instructions with an internal focus [60,61]. Therefore, in this study, subjects will be provided with generalised instructions regarding the task goals. Thus, for example, subjects will be given no specific instructions about weight shifting or control of the limb trajectory during stepping reactions, but will instead simply be told to take as few steps as possible or to avoid contacting the foam-rubber barriers. Where possible, subjects will be provided with an external focus, such as the handrail to be grasped or the foam blocks that they must avoid.

### Subject tolerance and acceptance

A pilot study was performed to evaluate the degree to which older adults with instability problems are willing and able to tolerate the training procedures. As described in more detail elsewhere (Maki et al., paper in review), the study involved eight older adults who were referred to a clinical falls prevention program, due to problems with instability, falling and/or fear of falling. Subjects were assigned, at random, to either the perturbation-based program or a program that focussed on training of rapid volitional stepping and grasping movements. The pilot results support the feasibility and safety of the perturbation-based program, and provide no evidence that balance-impaired older adults would be less able or willing to tolerate this approach in comparison to training of volitional movement. Compliance was very similar in the two groups. Moreover, no subjects indicated that they felt unsafe during the training, and there were no injuries or other adverse outcomes in either training program.

## Methods – Part B

### Experimental protocol to assess the training program

A randomised controlled trial will be performed to assess the efficacy of the perturbation-based training program. Ethical approval for this study was obtained from the Research Ethics Board of Sunnybrook Health Sciences Centre.

#### Recruitment of subjects

Community-dwelling older adults, aged 64–80 years, will be recruited via advertisements in local newspapers and seniors' residences and by word of mouth. All subjects will be mobile (not dependent on mobility aids) and will be able to stand (≥ 1 minute) and walk (≥ 10 m) without any assistance. To simplify training and assessment, the study will be restricted to persons who are right upper- and lower- limb dominant (as defined by the preferred limb for writing and kicking). In order to target an 'at risk' population, volunteers who respond affirmatively to any of the following questions will be included in the study:

• Have you fallen in the past 5 years?

• Have you lost your balance or almost fallen in recent memory?

• Have you reduced your activities or changed your lifestyle because you were concerned that you might fall?

• Do you ever feel unsteady when: getting in or out of a chair, changing direction when you are standing or walking, reaching for something above your head, or walking and talking to someone at the same time?

• Has anyone, such as a friend or family member, expressed concern that you may have problems with your balance or are at risk of falling?

Additionally, volunteers who report a decline in their balance greater than a 'moderate amount' in recent years, or have low balance confidence (Activity-specific Balance Confidence score less than 85% [62]), will be included in the study.

To avoid potential confounding effects on balance and to ensure a relatively homogeneous cohort, the following exclusion criteria will be applied: 1) current diagnosis of neurological or sensory disorders, diabetes, depression or osteoporosis; 2) uncorrected vision problems that impair ability to read, watch television, or drive a car; 3) recurrent dizziness; 4) cognitive impairment (standardized Mini-Mental State Examination [63] score < 24); and 5) joint replacement or joint fusion. To ensure that the subjects are sufficiently fit and healthy to tolerate the training program without adverse effects, volunteers who answer 'yes' to any items on the Physical Activity Readiness Questionnaire [64] will be asked to obtain approval from their doctor prior to participating in the study. To prevent potential confounding effects of other exercise programs, volunteers who regularly (≥ once per week) participate in a supervised exercise program will be excluded. Subjects who are unable to provide written informed consent (e.g. due to poor English language comprehension) will also be excluded.

#### Randomisation and blinding

Subjects will be randomly assigned to either the perturbation-based training group or a control group. Randomization will be stratified in blocks of two according to sex, age (64–72 or 73–80 years old), baseline compensatory stepping performance (average number of 'extra' steps required to recover balance: <1 or ≥ 1), and baseline compensatory grasping performance (reaction time: <120 ms or ≥ 120 ms). Random sequence generation will be performed by an individual who will not interact with subjects during the balance-testing sessions (AM).

Subjects will be informed that they will be randomly assigned to one of the two training programs, and will not be led to expect that either program will be more effective in improving their balance. The individual who administers the training programs (AM) will be the only member of the research team aware of the subjects' group allocation. A blinded research assistant will administer the balance tests and will perform any data processing that involves subjective judgements. Scripts will be used during testing to ensure that all subjects receive the same instructions.

#### Cohort descriptors

A number of measures will be collected for purposes of characterising the cohort, in terms of sensory, musculoskeletal, neuromotor and cognitive function, balance and mobility, anthropometrics, psychometrics, health status and lifestyle. As indicated in Table 2, a subset of these measures will also be used to compute an overall falls-risk score (FallScreen^© ^[65]), based on a database compiled from numerous prospective falls-prediction studies [66-69].

**Table 2:** Cohort descriptors

**General domain**	**Specific domain**	**Instrument**
Anthropometrics		Weight^†^, height, arm span, and waist and hip circumference

Sensation	Vision	High- and low-contrast visual acuity^§^
		Edge contrast sensitivity (Melbourne Edge Test)^§^
		Depth perception^§^
	Vibration sense	200 Hz vibration at the knee^§^
	Proprioception^†^	Joint position matching task, left and right knee angle^§^
	Touch sensitivity	Monofilament test at the plantar heel [83]
	Vestibular	Vertical X-Writing Test (proxy measure) [84]

Musculoskeletal	Isometric strength^†^	Ankle dorsiflexion^§^
		Knee extension ^§^
	Lower-limb power^†^	Repeated Step-Up (proxy measure) [79]
	Flexibility^†^	Sit-and-reach test [78]

Neuromotor	Coordination	Finger-to-nose touch test [85]
	Simple reaction time^†^	Finger press^§^
		Foot pedal^§^

Cognitive	Cognitive function	Standardized Mini-Mental State Examination (sMMSE) [63]
	Memory and recall	Supraspan forward digit recall with motor component [86]
	Visuospatial memory	Backward digit recall [87]

Balance & mobility	Static balance^†^	Spontaneous postural sway, measured at pelvis; four conditions: eyes open or closed, standing on firm surface or foam^§^
	Dynamic balance^†^	Coordinated stability^§ ^(volitional centre-of-mass movement around a designated course)
		Maximal balance range^§ ^(range of forward and backward lean)
	Mobility^†^	Timed Up and Go [77]

Health and lifestyle	General health	Medical Outcomes Study, Short form (MOS SF-36) [88]
	Falls history	Custom-designed questionnaire
	Exercise behaviour	Physical Activity Scale for the Elderly (PASE) [89]
	Activities of daily living	Instrumental Activities of Daily Living [90]

Psychometrics	Balance confidence^†^	Activity-specific Balance Confidence questionnaire (ABC) [62]
	Trait anxiety	Endler Multi-dimensional Anxiety Scale – Trait (EMAS-T) [91]
	State anxiety^†‡^	Endler Multi-dimensional Anxiety Scale – State (EMAS-S) [91]
	Depression	Centre for Epidemiologic Studies Depression Scale (CES-D) [92]
	Personality	Neuroticism Extraversion Openness Five Factor Inventory (NEO-FFI^TM^) [93]
	Fear of falling and activity restriction	Survey of Activities and Fear of Falling in the Elderly (SAFFE) [94]

^†^These tests will be completed pre- and post-intervention

^‡^This questionnaire is administered immediately following balance testing

^§^These tests are part of the FallScreen© assessment [65, 95]

#### Intervention

Each training program will last six weeks, with three 30-minute sessions per week. This duration and intensity is similar to that used in previous perturbation-based training studies [34,35,39]. All subjects will be asked to refrain from initiating any other new exercise programs, or otherwise consciously changing their activity levels, during their participation in the study. For the perturbation-based training program, each session will consist of 2–3 minutes of preparation and instruction, 10–12 minutes of step training, 3–5 minutes of rest, and 10–12 minutes of grasping training. We aim to complete at least 24 trials of stepping and 24 trials of grasping per session (three sets of eight trials for each).

The control subjects will undergo a flexibility and relaxation program, and will receive the same degree of interaction with experimenters as those in the perturbation-training group. The first session of each week will involve 30 minutes of passive muscular relaxation exercises [70]. The second and third sessions will focus on lower- and upper-body flexibility exercises, respectively. In the flexibility sessions, a ten-minute warm-up (heart rate kept below 60% of maximum) will precede 15 minutes of stretching exercises, (two sets of 7–9 stretches for each muscle group, with each stretch held for 15 seconds). The control program is designed to involve a limited intensity of physical activity, so as to avoid bringing about any significant physiological changes. A similar, but more intense, flexibility and relaxation program failed to reduce falling [9], or brought about only moderate improvements in falls risk [71]. Subjects will be asked not to practice the relaxation or flexibility exercises outside of the scheduled sessions during the study.

#### Testing of compensatory stepping and grasping reactions

Two balance-laboratory assessments will be performed to evaluate the effects of the training on compensatory stepping and grasping. The first session will occur not more than one week before the start of training and the second no more than one week after the end of training. Two different types of balance perturbation will be used: waist-pulls and support-surface translations. Because support-surface translations are also used during the perturbation-based training, testing with surface translations will allow us to identify whether the targeted aspects of the change-in-support reactions were improved by the training. The waist pulls involve perturbation of the centre of mass (rather than the base of support), elicit different patterns of body motion and sensory drive, and evoke stepping and grasping reactions that differ in some key respects in comparison to reactions evoked by platform perturbation [72,73]; hence, the waist-pull tests will allow us to begin to examine whether any of the benefits of the training are generalisable to other balance-loss situations.

**Figure 4:**
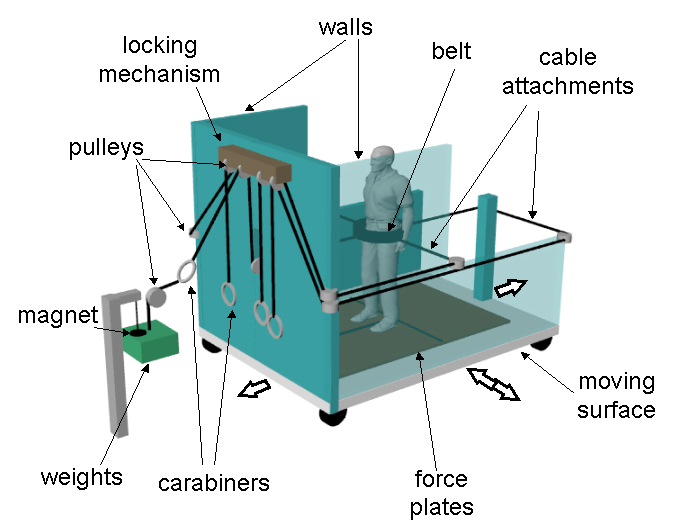
**Experimental set-up for testing of stepping and grasping reactions**. Schematic drawing showing the experimental set-up for the balance testing (performed at baseline and after the six weeks of training). The platform is semi-enclosed, with walls to the front and sides of the subject; for illustration purposes, the left wall has been rendered semi-transparent. The motion platform is controlled by a motor (located underneath the surface) to move unpredictably in one of the four directions shown. The cable-pull system also delivers unpredictable perturbations in these four directions. Four cables are attached to the belt at the waist, and are routed via a system of pulleys to a weight-drop apparatus that is located behind the front wall of the platform (out of the view of the subject). Prior to each trial, the experimenter manually connects one of the four cables to the weight. An electromagnet is then used to initiate the weight drop. When the weight is dropped, the subject is pulled unpredictably in one of the four directions, depending on which cable is attached to the weight. Prior to perturbation onset, an equivalent amount of slack (~2–4 cm) in each cable is maintained via a locking mechanism; hence, subjects are free to sway to an equal extent in any direction and cannot detect which cable is attached to the weight. During the testing of grasping reactions, a handrail (not shown) is mounted to the right of the subject (25% of body height from midline; height  of rail = 55% of body height) and foam blocks (40cm high) are placed around  the feet to deter stepping (similar to Figure 2B).

All laboratory tests will be performed with the subject standing at the centre of a large (2 × 2 m) computer-controlled motor-driven motion platform [17,74]. The waist-pull system is mounted on the motion platform, thereby allowing the type of perturbation to be varied unpredictably during testing (Figure 4). Waist-pull perturbations will be applied by dropping a weight (20% of body weight), attached to a belt worn around the pelvis via a custom-built cable and pulley system. Four cables are attached to the belt to allow perturbations to be delivered unpredictably forward, backward, left or right. The weight is dropped 40 cm for stepping trials and 30 cm for grasping trials. For the support-surface translations, we will use a displacement, velocity and acceleration of 0.18 m, 0.6 m/s and 2.0 m/s^2^, respectively, for forward translations (which evoke backward falling motion), and 0.27 m, 1.0 m/s and 3.0 m/s^2 ^for the other translation directions (backward, left, right). We know, from pilot tests and previous studies [22-24], that the selected waist-pull and surface-translation parameters are tolerated by older adults and will consistently evoke stepping or grasping reactions.

Perturbation direction and type (i.e. waist pull or surface translation) will be varied in an unpredictable pseudo-random sequence so as to prevent subjects from pre-planning their response. An initial set of 12 familiarisation trials will be completed at the start of each laboratory session in order to dampen within-session habituation effects [47]. Following this, three blocks of perturbation trials will be completed: 1) compensatory stepping during stance, 2) compensatory stepping while walking in-place, and 3) compensatory grasping during stance. Table 3 details the task conditions and numbers of trials completed in each trial block.

**Table 3:** Balance tests performed before and after training

**Task conditions**	**Response observed**	**Perturbations and numbers of trials**^†^
*Familiarisation*: Stance, unconstrained arm and foot motion (instructed to react naturally)	not applicable	4 medium translations^‡ ^(F,B,L,R) 4 large translations^‡ ^(F,B,L,R) 4 waist pulls (F,B,L,R) *Total *= *12 trials*

*Trial block #1:* Stance, arm motion restricted, instructed to react naturally but minimise number of steps	Compensatory stepping main focus: multiple-step responses (a-p perturbations)	5 backward translations 3 forward translations 2 lateral translations (L,R)^§^ 4 translations, 2^nd ^waveform (F,B,L,R)^#^ 5 forward waist pulls 3 backward waist pulls 2 lateral waist pulls (L,R)^§^ *Total *= *24 trials*

*Trial block #2:* Walking in-place, arm motion restricted, instructed to react naturally but minimise number of steps	Compensatory stepping main focus: foot collisions (lateral perturbations)	5 leftward translations 3 rightward translations 2 a-p translations (F,B)^§^ 4 translations, 2^nd ^waveform (F,B,L,R)^#^ 5 rightward waist pulls 3 leftward waist pulls 2 a-p waist pulls (F,B)^§^ *Total *= *24 trials*

*Trial block #3:* Stance, foot motion restricted, instructed to recover balance by grasping handrail (at right of subject)	Compensatory grasping main focus: speed of grasping (a-p perturbations)	5 forward translations 4 backward translations^§^ 4 forward waist pulls^§^ 5 backward waist pulls *Total *= *18 trials*

^†^During trial blocks #1–3, the listed platform-translation and waist-pull perturbations are delivered in an unpredictable randomised sequence, in the directions indicated (F = forward, B = backward, L = left, R = right; a-p = antero-posterior)

^‡ ^The familiarisation block includes initial medium-magnitude platform perturbations, followed by the same large-magnitude perturbations that are used in the main trial blocks. The platform acceleration of the large perturbations (2.0 m/s^2 ^forward, 3.0 m/s^2 ^other directions) is 50% greater than the medium perturbations (1.3 m/s^2 ^forward, 2.0 m/s^2 ^other directions).

^§ ^These tests are included primarily for the purpose of increasing the unpredictability of the perturbation direction and will not be part of the main analysis

^# ^The 2^nd ^waveform tests will not be part of the main analysis, but are included to deter subjects from learning to use the deceleration of the platform to aid in recovering balance [46]. The usual waveform comprises a 300 ms acceleration pulse followed immediately by a 300 ms deceleration pulse. The 2^nd ^waveform comprises a 200 ms acceleration pulse and a 400 ms constant-velocity interval, prior to a 200 ms deceleration pulse. The acceleration magnitude for this second waveform ranges from 1.35 m/s^2 ^to 2.25 m/s^2^.

During compensatory stepping tests, subjects will be asked to respond to the balance perturbations naturally, but to minimise the number of steps taken if they do step. To restrict arm movement, they will hold a lightweight rod behind their back [74]. To deter conscious efforts to pre-plan or modulate the reactions, subjects will be required to perform a concurrent distraction task (counting backward by 3's from a 3-digit number randomly assigned at the start of the trial) while waiting for the perturbation, during the stance trials (blocks 1 and 3). In the second block of trials, they will perform a walk-in-place task, (lifting the feet in time with a metronome, 100 beats per minute) to increase the probability that a foot collision will occur [22]. For compensatory grasping trials, a handrail will be mounted to the right of the subject. Subjects will be instructed to grab the rail with the right hand as quickly as possible to recover balance, in response to the perturbation. They will be told not to step, and foam blocks will be placed around their feet to further deter stepping. A safety harness will be worn throughout all of the testing.

#### Outcome measures

The four primary outcome measures correspond to the specific aspects of change-in-support reactions that were targeted in the perturbation-based training program: 1) average number of 'extra' steps taken to recover balance, 2) frequency of foot collisions when responding to lateral perturbations, 3) frequency of additional lateral steps when responding to antero-posterior perturbations, and 4) handrail-contact time for grasping reactions. Secondary outcome measures relating to other features of the balance reactions will also be analysed. For compensatory stepping, these include the frequency and magnitude of anticipatory postural adjustments (APA), foot-off time, foot-contact time, step length and frequency of arm reactions. For compensatory grasping, we will analyse onset timing and magnitude of arm-muscle activation (biceps, medial deltoid), arm trajectory, and frequency of grasping errors (e.g, undershoot, overshoot, collision of hand with rail). The early 'automatic postural response' (which occurs in both stepping and grasping trials) will be analysed using centre-of-pressure measures and the latency and magnitude of the initial ankle-muscle activation (tibialis anterior, medial gastrocnemius).

The kinematic measures (step length, arm trajectory) will be determined by using a six-camera infrared motion-analysis system to record displacement of reflective markers placed bilaterally on the feet and arms. The behavioural measures (foot collisions, additional steps) will be determined using a computer-based algorithm to process the foot-marker kinematics; results will be verified by manual inspection of these data and associated video recordings by a blinded investigator. In addition, effectiveness of the stepping and grasping reactions in preventing "falls" will be analysed by using a load cell to monitor the force applied to the safety harness. Step timing, APAs and centre-of-pressure will be characterized using three force plates built into the surface of the motion platform. Handrail-contact timing will be determined from force-sensing resistors mounted on the rail. Muscle activation will be recorded bilaterally using surface electromyography (EMG). All timing measures will be defined in relation to onset of platform motion (measured with accelerometers) or onset of weight-drop (measured with a load cell). See previous articles for more details about the measurement and analysis methods [74-76].

Other secondary outcome measures will be analysed to evaluate whether the training had any benefits extending beyond effects on balance reactions. We will explore indirectly the effect on overall falls risk using the FallScreen^© ^battery noted earlier. In addition, we will explore effects on: 1) balance confidence (Activity-specific Balance Confidence questionnaire [62]); 2) mobility ('Timed Up & Go' test [77]); 3) flexibility (modified 'sit and reach' test [78]); and 4) lower-limb power (using the repeated step-up test [79] as a proxy measure [80]).

#### Statistical analysis

The effect of the two training interventions on the outcome measures will be determined using repeated-measures analysis of variance (ANOVA) with two factors: 1) group (perturbation-based training versus control group), and 2) session (pre- versus post-training). Any of the cohort descriptors (measured at baseline, as detailed earlier) that show significant inter-group differences in preliminary univariate analyses will be included as covariates in the main analysis. As noted earlier, the control training program was specifically designed not to improve balance control; therefore, changes in the control group (pre- versus post-training) are expected to represent typical learning effects resulting from repeated exposure to the balance-assessment procedure, as well as possible 'placebo' effects associated with the interventions. Analysis of the group-by-session interaction will therefore reveal any real benefits resulting from the perturbation-based training intervention.

#### Sample size estimate

To date, five subjects have completed the perturbation-based training and five have completed the control intervention. Sample size calculations for repeated measures ANOVA [81] were performed using the variance from these initial ten subjects. Separate calculations were performed using two of the primary outcome measures, so as to determine the sample-size requirements based on stepping reactions (average number of 'extra' steps taken to recover balance) and grasping reactions (handrail-contact time). For both calculations, probability of Type I error was 0.05, and probability of a Type II error was 0.2.

A previous study of older adults showed that the average number of 'extra' steps taken to recover balance was 1.21 in 'fallers' and 0.825 in 'non-fallers' [25]. This suggests that a reduction equal to 0.5 steps, due to the perturbation-based training, will have functional significance. Using this value, in combination with the initial variance estimate (SD = 0.44 steps), it was determined that 15 subjects per group will be required. For grasping responses, the previous study [25] indicated that handrail-contact time was delayed by 50 ms, on average, in the 'fallers'. Detection of a training effect of this size, given the initial variance estimate (SD = 39 ms), requires a sample of only 12 subjects per group. Thus, to ensure sufficient statistical power for both stepping and grasping measures, it appears that 15 subjects per group will be required. However, sample-size requirements will be re-evaluated at two intermediate stages, i.e. after completing 10 and 15 subjects in each group. If any subjects are still enrolled in either training program when the new estimate for the required number of subjects is reached, these remaining subjects will complete the study and the tested sample will exceed the estimated requirement.

## Discussion

This study is among the first to use perturbation-based training as an intervention to reverse age-related impairments in the ability to recover from sudden loss of balance using rapid stepping or grasping reactions. It differs from the previous studies in this area in a number of significant ways. To date, four studies have used perturbations to train stepping reactions; however, two of these focussed on Parkinson's Disease [35,39]. The other two studies showed potential to improve volitional reaction time in older adults [34,36]; however, they did not assess effects of the training on compensatory stepping. No study to date has investigated the potential to use perturbations to train compensatory grasping reactions.

The perturbation-based training program outlined in this protocol is believed to improve on previously proposed programs because: 1) it targets specific aspects of balance-recovery reactions that are known to be impaired in older adults and associated with increased falling risk; 2) the perturbations challenge balance control in multiple directions; 3) the perturbations are unpredictable and hence prevent adaptations that rely on predictive control; 4) the perturbations are applied in a manner that allows for well-controlled progressions in perturbation magnitude; 5) ongoing cognitive and movement tasks are included to increase the generalisability of training; and 6) the training methodology (use of instructions, feedback, etc) adheres to principles that are known to promote optimal motor learning.

We believe that these features will increase the probability that the program will bring about functionally significant improvements in the control of compensatory stepping and grasping. Ultimately, such improvements should help to reduce the incidence of falls. We plan to collect preliminary falls data by monitoring falling in our subjects for a one-year period following the completion of training (using monthly report cards and telephone follow-up [41]); however, investigation of effects on falling is beyond the intended scope of this initial study, and the current sample will likely be too small to determine significant changes in fall rate.

An important factor that may strongly influence the longer-term benefits of the training is the inclusion of some form of maintenance program, which will likely be needed to prevent reversal of the training benefits [82]. Such a maintenance program could potentially involve exercises that can be performed at home, without supervision; however, the development and testing of such a program is beyond the scope of this initial study.

The results of this study, and the initial results from the falls follow-up, will provide new evidence regarding the effectiveness of perturbation-based balance training programs, which may in turn justify additional studies to examine the generalisability of the training benefits to a wider range of balance perturbations and balance-loss situations, as well as larger-scale studies to examine the effects of the training on risk of falling and fall-related injury. If the results are promising, then future efforts will be directed at developing and testing simpler and less expensive methods for administering the perturbations during training, as well as cost-effective exercise programs for long-term maintenance of the training benefits. We anticipate that these efforts will help to guide the development of more effective falls prevention programs, which may ultimately lead to reduced health-care costs and enhanced mobility, independence and quality of life.

## Competing interests

The author(s) declare that they have no competing interests.

## Authors' contributions

All authors contributed to design of the study and the development of the balance training program. AM wrote the paper, and will administer the training program, collect the data, process the data that do not require blinding, and perform the statistical analyses. ALP will assist with data collection, processing and analysis. BL and BEM are the study co-principal investigators who devised the study and will provide guidance and supervision during the data collection, processing and analysis. All authors contributed significantly to the preparation of the paper, and read and approved the final version.

## Pre-publication history

The pre-publication history for this paper can be accessed here:


